# The critical role of parents within a Learning Health Network

**DOI:** 10.3389/fped.2024.1428758

**Published:** 2024-08-09

**Authors:** Kerry Ferraro, Jenny Leal, Anna Sutton, Susan Peters, Corinne Pinter

**Affiliations:** ^1^ JIA Parent and Pediatric Rheumatology Care and Outcomes Improvement Network Volunteer, Lower Gwynedd, PA, United States; ^2^ JIA Parent and Pediatric Rheumatology Care and Outcomes Improvement Network Volunteer, Columbus, OH, United States; ^3^ JIA Parent and Pediatric Rheumatology Care and Outcomes Improvement Network Volunteer, Woodinville, WA, United States; ^4^ JIA Parent and Pediatric Rheumatology Care and Outcomes Improvement Network Volunteer, Cypress, TX, United States; ^5^ JIA Parent and Pediatric Rheumatology Care and Outcomes Improvement Network Volunteer, Sugar Land, TX, United States

**Keywords:** engagement, parent, partners, juvenile arthritis, quality improvement, pediatric rheumatology, Learning Health Network, co-production

## Abstract

Parent members of the Pediatric Rheumatology Care & Outcomes Improvement Network are an integral part of the Learning Health Network's work. Since early in the creation of the network, they have been a part of every Quality Improvement project, committee, and work group and have a role in governance on the Executive and Steering Committees. Members of the Parent Working Group (PWG) have played a role in developing QI measures used in the clinical setting as well as initiatives and projects like the guiding work of Treat-to-Target. The PWG also creates self-management supports, including toolkits for families and patients at all stages of life. This article will discuss how integrating parents as partners in a pediatric Learning Health Network is critical for the quality of care received by children with chronic illnesses and to improving outcomes.

## Introduction

Parent members of the Pediatric Rheumatology Care & Outcomes Improvement Network (PR-COIN) have been an integral part of the Learning Health Network since soon after its formation in 2010. As early as 2012, PR-COIN invested resources to build capacity for engaging families at its centers. A Parent Engagement Consultant was hired to create and accelerate strategies for participation and engagement of families at both individual member team and PR-COIN network levels. In 2014, parents were invited to join Committees, including the Steering Committee, and funding was provided for three parents to attend the Learning Session.

The role of the parents took off in 2015 when the parent group chose to name itself the Parent Working Group (PWG) and to better define their mission and objectives within PR-COIN. PR-COIN added family engagement to its center agreements. Parents co-presented with providers at the Learning Sessions, and created toolkits and resources. The Engagement Committee was created, with this article's co-author Kerry Ferraro being named as the lead. She also became the first parent to join the Executive Committee in 2019. Since then, PR-COIN has enshrined patients with Juvenile Idiopathic Arthritis (JIA) and their families in its approach and its mission “to build a thriving and inclusive community of patients, families, clinical teams and researchers that uses quality improvement (QI) science to deliver exceptional and equitable health care to children with rheumatic diseases and to bring research discovery to patients faster.” This approach involves including patients and families in all levels of governance and network activities to ensure patient-centered care. Parents are partners in the work (1).

**Figure 1:**
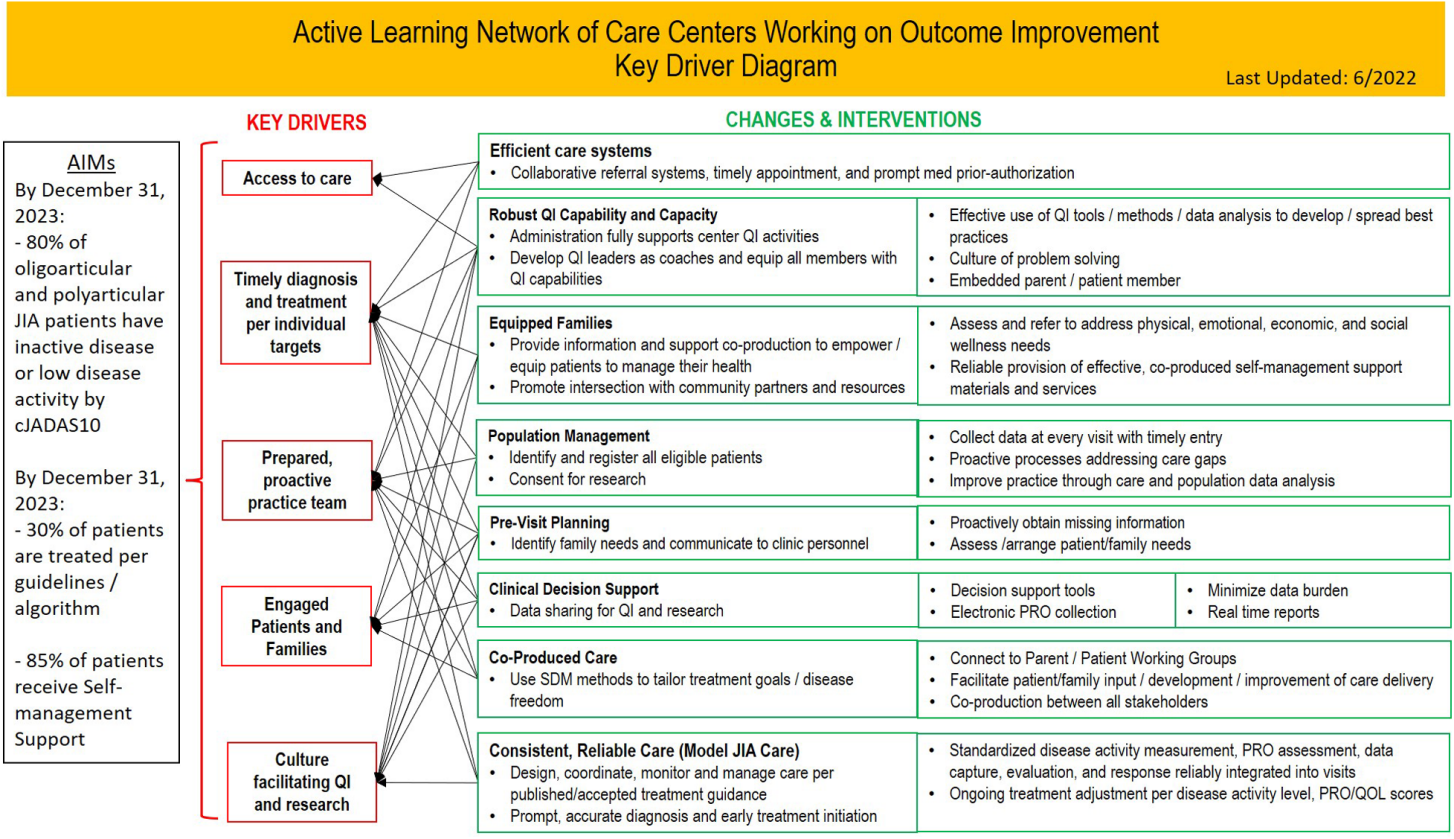
PR-COIN's key driver diagram. Reprinted with permission from Pediatric Rheumatology Care and Outcomes Improvement Network (PR-COIN), licensed under Creative Commons BY-NC-ND, https://www.pr-coin.org/s/PR-COIN-Key-Driver-Diagram.png.

PR-COIN's Key Driver Diagram, shown in Figure 1, identifies Engaged Patients and Families as a crucial key driver. Several of the changes and interventions explicitly emphasize parent engagement, such as Embedded parent/patient member; Connect to Parent/Patient Working Groups; Facilitate patient/family input/development/improvement of care delivery; and Co-production between all stakeholders. Moreover, parents have significantly contributed to the implementation of other changes and interventions outlined in the Key Driver Diagram.

A key member of PR-COIN's leadership team, Dr. Julia Harris wrote in her paper, “Improving Care Delivery and Outcomes in Pediatric Rheumatic Diseases” (2): “Engaging patients and families is an increasingly recognized component for quality improvement in healthcare that is patient centered. PR-COIN has formed a parent leadership roundtable, Facebook page, parent attendance at action period calls, and robust parent presence at learning sessions. Parents set 90-day goals, hold positions at leadership level and on PR-COIN subcommittees, and contribute novel talent and skills to the network. Through patient/parent engagement, PR-COIN is striving to foster co-production of network activities whereby health care teams and patients/families can work together to produce more desirable outcomes for children with JIA.”

Beyond the governance and co-production areas, parent support extends to the organization's fiscal health. Parents are key members of the Finance and External Partnerships Committee and have written letters to hospital leadership to encourage them to join or remain a member of PR-COIN. They have been involved in the creation of marketing and fundraising materials, held fundraisers, and assisted in securing grants. This not only supports the organization, but it also eases the financial burden on the centers.

Parents and families are an integral part of the guiding work and ability of PR-COIN to achieve QI. This article will discuss the critical role of parents in implementing the changes and interventions that support the key drivers of improvement within a pediatric Learning Health Network by exploring examples of parent involvement in four specific interventions under the categories of Robust QI Capability and Capacity, Equipped Families, Co-Produced Care, and Robust, Reliable Care. In this article, “parent” is being used to refer to the primary caregiver.

### Key driver diagram

PR-COIN's Key Driver Diagram (KDD), shown in Figure 1, identifies the network's AIMS as of December 31, 2023 and highlights the key drivers of Access to care; Timely diagnosis and treatment per individual targets; Prepared, proactive practice team; Engaged Patients and Families; and Culture facilitating QI and research. The changes and intervention categories on the right of the KDD are Efficient care systems; Robust QI Capability and Capacity; Equipped Families; Population Management; Pre-Visit Planning; Clinical Decision Support; Co-Produced Care; and Consistent, Reliable Care (Model JIA Care). We will explore specific interventions that fall under four of these categories.

### Robust QI capability and capacity

Within the Robust QI Capability and Capacity category, the intervention “Embedded parent/patient members” explicitly emphasizes parent engagement. Additionally, voices of parents are heard at the very core of PR-COIN's work—the development of the quality improvement measures used to track patient outcomes under the Effective use of QI tools intervention. As shown on the KDD, these interventions support each key driver of the network.

Dr. Catherine Bingham's paper “Pediatric Rheumatology Care and Outcomes Improvement Network's Quality Measure Set to Improve Care of Children With Juvenile Idiopathic Arthritis” (3) notes how each center forms a local QI team and states, “Because the voices of patients and families are invaluable to inform the challenges to care and impact of disease, patient/parent representatives are also included.”

A formal PR-COIN Measures Committee was formed in 2013 and was composed of volunteers from center teams and JIA parent representatives. Bingham discussed how the original Quality Measures (QM) were based on a 2008 ACR project to identify QMs for JIA that included surveys of pediatric rheumatologists, APNs, patients, and parents. This was also discussed by Dr. Daniel Lovell et al. in the 2011 paper, “Measuring Process of Arthritis Care” (4).

Parents were also involved in developing the new QM set in 2018 (3). Parents conveyed the importance of including the Patient Global Assessment of overall well-being and emphasized that the provider assessments alone do not capture the lived experiences of youth where, for example, medication reactions might leave a child in bed for days. Therefore, the percentage of patients who had a Patient Global Assessment of overall well-being less than or equal to two was added to the revised QM set by the PR-COIN Measures Committee (3).

Parent and patient voices are especially critical because often there is a discordance between providers and patients in disease assessment, which is the reason parents pushed for the Patient Global Assessment to be included in our QM set (5).

### Equipped families

Within the Equipped Families category, parents played a significant role in the interventions of: Provide information and support co-production to empower/equip patients to manage their health and Reliable provision of effective, co-produced self-management support materials and services. These directly support the key drivers of Timely diagnosis and treatment per individual targets and Engaged Patients and Families.

Disease outcomes are impacted heavily by a patient and their parent being informed and able to manage the condition effectively. Self-management activities include managing medications, whether injectable, oral or intravenous, and managing appointments as well as blood work, imaging and home exercise programs. It further extends to the daily life of the youth living with the disease, including going to school, managing health insurance coverage, and managing mental health. Self-management support (SMS) is one of the six essential elements of care identified in the CCM (6). Rheumatology nurse practitioner and former Outcome Committee co-lead, Janalee Taylor championed PR-COIN's SMS initiatives and believes “These activities must be integrated into daily life with consideration for developmental, intellectual, and psychosocial well-being of the child and family” (7).

Parents were included in the work groups for each of the tools created as part of the SMS system, which included the Barriers Assessment, Adherence Tools, Self-Management Assessment, and Helping Hands Handbook. Parents also co-presented on the tools at a Learning Session and encouraged providers to use the tools.

Member centers are provided with a SMS System Change Package. This is a toolkit to support practitioners and families in formulating and adhering to medication and treatment plans. The change package includes provider training in Behavior Change Counseling, and use of Self-Management Assessment Tool, Barriers Checklist, Adherence Solution Tools, Patient Action Plan worksheet, and complete family educational materials in the Helping Hands Handbook (8).

The Self-Management Assessment identifies patient/family priorities for visit, level of confidence in ability to manage disease, level of worry, goal setting, adherence and general barriers to care. The Barriers Checklist helps providers identify what comes in the way of following treatment. For each barrier, an adherence solution is identified, including corresponding tools and templates. The Helping Hands Handbook contains information about JIA, medications, treatments, and activities to optimize quality of life.

The Barriers Assessment provides a good example of how these toolkits are developed (9). Providers and parents worked together through an iterative process to design the Barriers Assessment Tool to screen for adherence barriers across 4 treatment modalities (i.e., oral medications, injectable medications, infusions, and physical/occupational therapy). This tool was initially implemented in seven rheumatology clinics across the United States and patient responses were collected for analysis. Seventy-seven percent (*n* = 444) of caregivers and 70% (*n* = 69) of patients reported at least one adherence barrier (9). Identifying the barriers enabled care teams to provide the tools needed to overcome the barriers to adherence.

Equipping families expands beyond the tools created as part of the SMS tools. The PWG identifies and creates tools and resources to assist patients and their families in navigating life with JIA. Navigating school with JIA is often a challenge, so we adapted ICN's Accommodation Toolkit, which provides information on ADA laws and academic, workplace, and public accommodations for patients with JIA. Five parents helped, but parent Laura Bouslaugh's expertise as a Civil Rights Specialist was especially helpful. Next, parents teamed up with young adult patients and created a College and JIA toolkit, which provides information on finding a “good fit,” preparing to go to college with JIA, tips for being successful in college with JIA including accommodations and how to request them, taking time off college, and personal perspectives of JIA patients.

This process is iterative. We learned from center feedback that the Helping Hands Handbook was not being provided to patients at all centers, so we created a one-page frequently asked questions resource that includes a QR code to the handbook. This allows centers to only print a single page rather than a 127-page handbook. We also created a one-page accommodations summary that links to the 23-page accommodations toolkit. Next, we created the insurance toolkit, which helps families navigate health plans, including prior authorizations, step therapy, and denied claims. We surveyed parents to identify what educational materials were needed at the time of diagnosis to identify gaps. One third of the respondents wrote in that they wanted information on ways to connect with other patients and families. We created a resource list for families that includes links to the PR-COIN toolkits, Arthritis Foundation resources, support groups, social media resources, and podcasts.

### Co-produced care

Co-producing care calls for including parents in the co-production of the care and the improvement work of the network under the interventions of: Connect to Parent/Patient Working Groups; Facilitate patient/family input, development and improvement of care delivery; and Co-Production between all stakeholders. It also calls for the Use of SDM (shared decision making) methods to tailor treatment goals and disease freedom.

PR-COIN follows the Chronic Care Model which states that quality care involves productive interactions between informed, activated patients and their care teams (6). To improve these interactions, one of PR-COIN's early interventions was the use of SDM methods. PR-COIN's Medication Choice Cards help improve parent and patient engagement and facilitate the discussions required for SDM.

**Figure 2:**
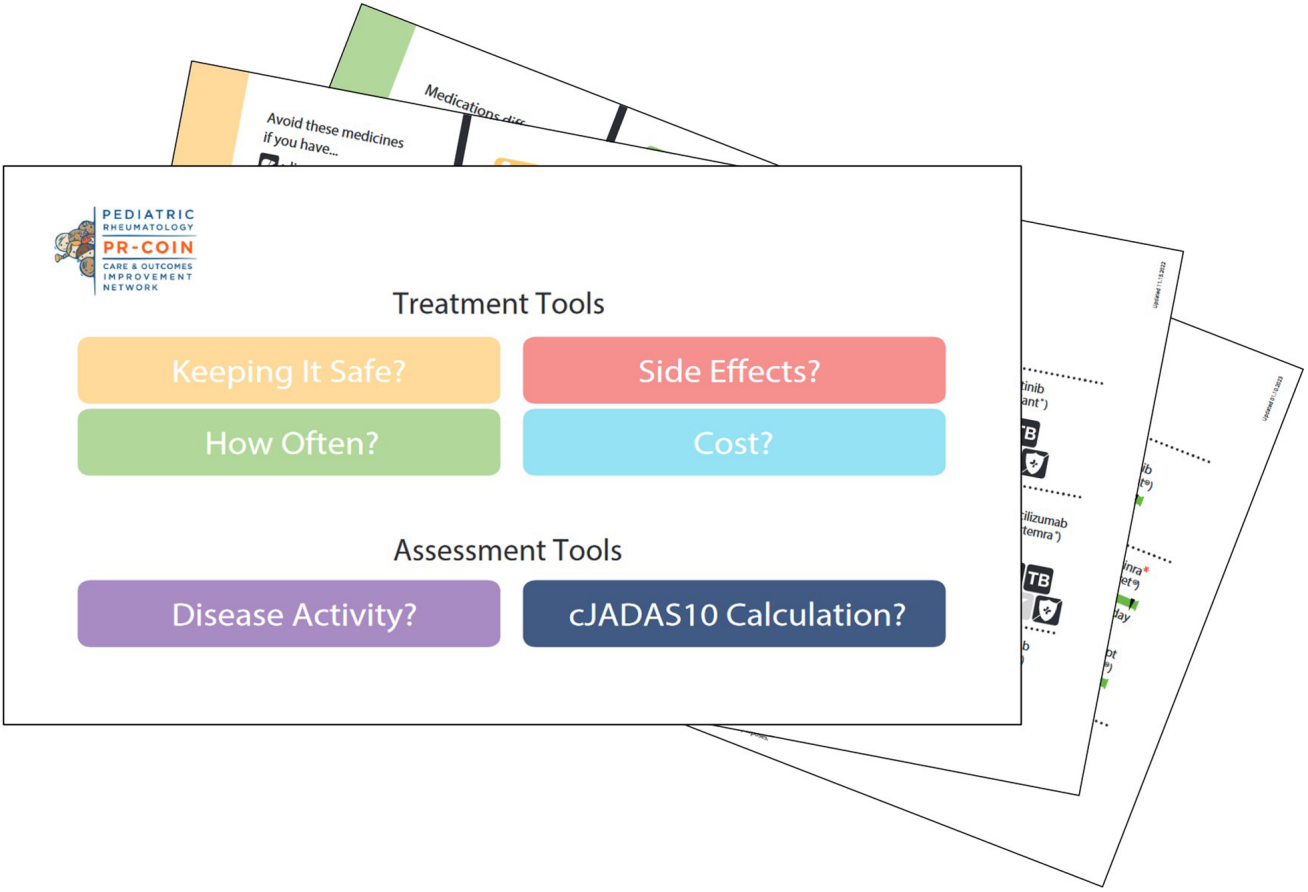
Sample image of PR-COIN's medication choice cards.

Parents played a role in the development and revisions of Medication Choice Cards, shown in Figure 2. These cards walk parents and patients through any choices they may be facing, allowing them to be informed and involved. The initial design of the cards involved an iterative process with a stakeholder panel of parents and care team members (10). Providers and parents co-presented the use of the Medication Choice Cards at a PR-COIN Learning Session. Six sites volunteered to use QI methods to implement the cards. Four of these sites collected parent surveys following visits to assess outcomes. Patients and parents shared on clinician use of the cards and the amount of SDM and uncertainty they experienced (11). The study authors concluded that more reliable use and sharing of best implementation practices was needed and questioned whether an electronic format may lead to more reliable use. In response, Ferraro created an interactive, digital format of the cards for use by clinicians. Another case study showed that use of the “Medication Choice Cards significantly enhanced shared decision making in treatment of adolescents with JIA, through increased patient engagement” (12).

As treatment options change, parents continue to partner in the updates and revisions to the paper and digital cards as well as to the pamphlet that was developed for parents to make notes on and take home to discuss the treatment choices with other family members.

### Consistent, reliable care

The key drivers include: Timely diagnosis and treatment per individual targets; Prepared, proactive practice team, and Engaged patients and families. To support these, PR-COIN identified the need for Consistent, Reliable Care as an intervention category. Due to its focus on all of the interventions under the Consistent, Reliable Care category on the KDD in Figure 1, Treat-to-Target (T2T) has become the foundational work of PR-COIN.

Dr. Sandy Burnham started T2T with polyarticular JIA and included a polyarthritis parent in the project meetings. Work on T2T began in 2004 when it was noted that not enough patients were achieving inactive disease. T2T takes the core concepts of measure, standardize, and use of clinical decision support and applies them in the clinical setting. As Burnham developed the project, he shared the patient-facing materials with parents for feedback. When PR-COIN decided to make the project network-wide, parents were partners in creating the educational materials for providers and families. “Designing and Testing Treat to Target as a New Care Model in JIA Across a Network of Pediatric Rheumatology Centers” notes that “With patient/family partners, PR-COIN co-produced educational materials to train providers on implementation of T2T and to introduce families to the concept…. Co-producing support materials with families, infrastructure to support QI, and reliable data submission are key to success” (12).

In 2020, PR-COIN held a consensus conference to standardize treatment plans (14). “PR-COIN stakeholders, including health care providers (*n* = 16) and parents (*n* = 4), were invited to form a voting panel.” When identifying the most important elements when setting an individual's target, parents advocated for patient goals. After the second round of voting, “patient goals” was voted unanimously as the most important element. Parents revised the Medication Choice Cards to include the clinical Juvenile Arthritis Disease Activity Score (cJADAS) calculation and disease activity level by JIA subtype to help identify if the disease level is at target. Since the cJADAS includes both the Physician Global Assessment and the Patient Global Assessment of wellbeing, this gives the clinicians the opportunity to discuss any discordance between the clinician's assessment of disease activity and the parent's or patient's assessment.

All these tools create a clinical practice that involves parents. In one example provided by a provider, an 18-month-old patient came to her with polyarticular JIA and very sick. The PR-COIN method of approaching the mom from her perspective and using tools like shared decision making and T2T intervention helped the mom gain more acceptance of the treatment. Her anxiety level came down as the child was doing better and as she understood options and saw that labs helped monitor safety (15).

## Discussion

Beginning with early investments in parent engagement and the establishment of the Parent Working Group, PR-COIN has recognized and integrated the invaluable insights of parents into its governance and improvement initiatives. This collaborative approach has enriched the network's strategies and has empowered families.

The involvement of parents in pivotal roles such as committee membership, co-presentation at learning sessions, and co-production of essential tools and interventions underscores the impact of parents on care delivery and patient outcomes. Including parents as partners in a pediatric learning health network (LHN) fosters a deeper level of engagement with parents and patients during clinic visits and leads to a more patient-centered approach. PR-COIN's commitment to patient-centered care is evident in how parents and patients are integrated at every level of Key Drivers and Changes and Interventions. By championing shared decision making and advocating for patient goals in treatment planning, parents have fostered a culture of trust and collaboration between healthcare providers and families.

The impact of parent involvement in PR-COIN extends beyond clinical settings, influencing network governance, financial sustainability, and outreach efforts aimed at expanding PR-COIN's impact. PR-COIN parents are currently leading the development of an engagement measure to better evaluate how centers engage parents in their work, aiming to quantitatively assess the level of engagement and its impact outcomes of QI initiatives.

Looking ahead, future studies utilizing this engagement measure could provide insight on the correlation between parent engagement and improved health outcomes within pediatric LHN. Other LHNs who wish to improve parent and patient involvement in healthcare decision-making and network governance would do well to look at PR-COIN as a model.

## Data Availability

The original contributions presented in the study are included in the article/Supplementary Material, further inquiries can be directed to the corresponding author.
